# Synergistic Antimicrobial Activity of Biogenic Silver Nanoparticles and *Acanthospermum australe* Essential Oil against Skin Infection Pathogens

**DOI:** 10.3390/antibiotics13070674

**Published:** 2024-07-20

**Authors:** Javier Mussin, Gustavo Giusiano

**Affiliations:** Instituto de Medicina Regional, Universidad Nacional del Nordeste, Consejo Nacional de Investigaciones Científicas y Tecnológicas (CONICET), Resistencia 3500, Argentina; gustavogiusiano@yahoo.com.ar

**Keywords:** silver nanoparticles, essential oils, natural products, antimicrobial activity, synergistic effect, skin infections

## Abstract

In response to the steady increase in antimicrobial-resistant strains, the World Health Organisation has emphasised the need to investigate new antimicrobial agents and alternative therapies that improve the spectrum of activity and reduce the dose required, thus improving safety. This study focused on the characterisation of *Acanthospermum australe* essential oil and green-synthesis silver nanoparticles (AgNP), evaluating their cytotoxicity in human cells, antimicrobial activity and synergistic effect against pathogens causing skin infections. The main components of the essential oil were germacrene A (24.07%), γ-cadinene (21.47%) and trans-caryophyllene (14.97%). Spherical AgNP with a diameter of 15 ± 3 nm were synthesised. The essential oil showed antimicrobial activity against dermatophytes and *Malassezia globosa*, while AgNP were found to be active against bacteria, yeasts and dermatophytes. Both compounds were found to be primarily non-cytotoxic at the concentrations required to inhibit microbial growth. Furthermore, the combined use of essential oil and AgNP showed a synergistic antimicrobial effect against dermatophytes and *M. globosa*. In conclusion, the results suggest that the combined use of bioactive compounds from natural sources, such as essential oil and biogenic AgNP, has the potential to improve antimicrobial efficacy against specific skin pathogens, particularly *Microsporum canis*, *Nannizzia gypsea* and *M. globosa*.

## 1. Introduction

The skin is a vital barrier between the human body and its environment. This physical and biochemical barrier, formed by multiple layers of epithelial cells, glandular secretions, and a highly regulated microenvironment, is the first line of defence of the immune system and plays an essential role in protecting against microbial infections. However, despite its effective defence mechanism, the skin is susceptible to a wide range of infections caused by microorganisms that can penetrate its protective barriers. These infections, known as skin and soft tissue infections, encompass a wide range of clinical conditions from mild superficial to serious and life-threatening diseases [1,2].

Skin infections are caused by a wide spectrum of microorganisms that can colonise the skin and underlying structures and take advantage of favourable conditions for proliferation and survival. Among the most common bacteria associated with skin infections are *Staphylococcus aureus*, a Gram-positive bacterium that causes superficial infections to serious invasive disease, and *Streptococcus pyogenes*, responsible for conditions such as cellulitis and impetigo. In addition, *Pseudomonas aeruginosa* and *Escherichia coli*, opportunistic Gram-negative bacteria, can also play a role in certain skin infections, particularly in hospital settings [1,2].

Fungal skin infections caused by yeasts of the genus *Malassezia* and *Candida*, and dermatophytes (*Epidermophyton*, *Microsporum*, *Nannizzia* and *Trichophyton*), are also a significant public health problem, particularly in vulnerable populations such as immunocompromised patients, diabetics and the elderly. These microorganisms can colonise the skin and skin appendages and cause fungal conditions such as ringworm, cutaneous candidiasis and pityriasis versicolor, which can be difficult to treat and recurrent [1,2,3,4,5].

In response to the increasing incidence of skin infections and the emergence of antimicrobial-resistant strains, the World Health Organisation (WHO) has emphasised the importance of research and development of new antimicrobial drugs [6,7]. In this context, nanotechnology has emerged as a promising tool for the development of innovative antimicrobial agents. Silver nanoparticles (AgNP) have attracted considerable attention due to their unique antimicrobial properties, enabling them to combat a wide range of pathogens, including bacteria, yeasts and filamentous fungi. AgNP act through multiple mechanisms, including the controlled release of silver ions that interfere with the cellular functions of microorganisms, such as DNA replication and cellular respiration [8].

Silver has been used for therapeutic purposes since ancient times, as evidenced by traditional Indian medicine (Ayurveda), to treat various conditions, including skin infections and wounds. This ancient practice reflects the historical recognition of silver’s antimicrobial and anti-inflammatory properties in the treatment of infectious diseases [9,10]. On the other hand, medicinal plants have played a prominent role in ethnomedicine, being used by different cultures as a source of bioactive compounds with therapeutic properties [11]. Among these, the traditional use of *Acanthospermum australe* (Loefl.) Kuntze, a plant used by the indigenous peoples of South America to treat a wide range of ailments, including skin infections, stands out [12,13].

In this context, the present study aims to investigate the therapeutic potential of *A. australe* essential oil and a solution of AgNP obtained from an aqueous extract of *A. australe* against bacteria and fungi that cause skin infections. In addition to assessing their antimicrobial activity, we also evaluated their cytotoxicity in human cells and the synergistic effects between these compounds. This integrated approach provides a solid foundation for future research using appropriate in vivo models to develop new, effective and safe antimicrobial therapies for the treatment of skin infections, addressing a pressing medical need in the modern era.

## 2. Results

### 2.1. Gas Chromatography–Mass Spectrometry (GC-MS) Analysis of Essential Oil

GC–MS allowed the identification of the main components of the essential oil obtained from the leaves of *A. australe*. The total chemical composition is given in Table 1 and the total ion chromatogram recorded by GC–MS of the essential oil is shown in Figure S1.

The main constituents of the essential oil of *A. australe* leaves were germacrene A (24.07%), γ-cadinene (21.47%) and trans-caryophyllene (14.97%). These constituents represented more than 60% of the total constituents. Each of the other compounds was found in a percentage of less than 6% in the essential oil.

### 2.2. Synthesis and Characterisation of Silver Nanoparticles

**Figure 1:**
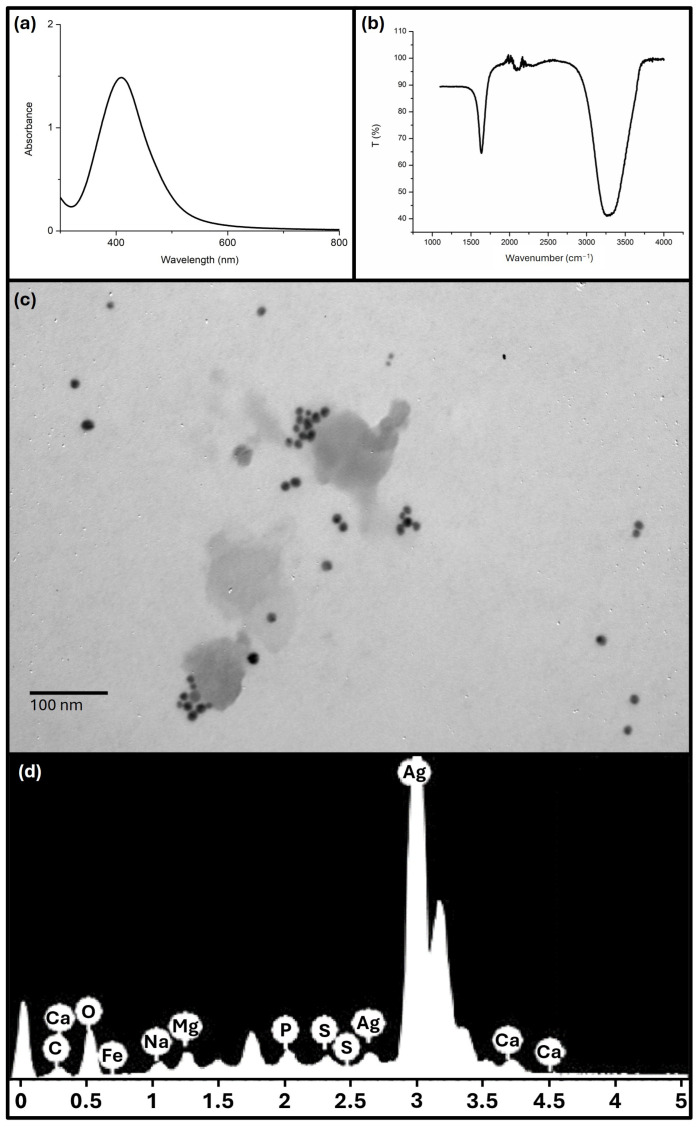
(**a**) UV–visible spectrum, (**b**) FTIR, (**c**) TEM image and (**d**) EDX of the synthesised AgNP.

During the green synthesis process, the colour of the solution changed from light yellow to dark brown within a few minutes, indicating the successful formation of AgNP. Figure 1 shows the results of the characterisation of the synthesised AgNP. The UV–visible spectrum manifests a maximum absorption band at 411 nm, corresponding to the typical surface plasmon resonance of AgNP. TEM images showed non-agglomerated spherical nanoparticles with a diameter of 15 ± 3 nm. EDX analysis revealed a strong elemental silver signal with an optical absorption band peak in the range of 3 to 4 keV, typical of the absorption of silver nanocrystals. Other elemental signals were recorded at lower levels. In the FTIR spectrum of the AgNP, peaks around 1636, 2100 and 3300 cm^−1^ were observed. DLS analysis revealed highly anionic monodisperse nanoparticles with a polydispersity index of 0.270, a hydrodynamic diameter of 35 ± 14 nm and a zeta potential of −35 ± 6 mV. In addition, the colloidal AgNP solution remained stable for more than 30 days without precipitation or colour change, demonstrating the excellent stability of the synthesised AgNP.

### 2.3. Cytotoxicity Assay of the Essential Oil and Silver Nanoparticles

**Figure 2:**
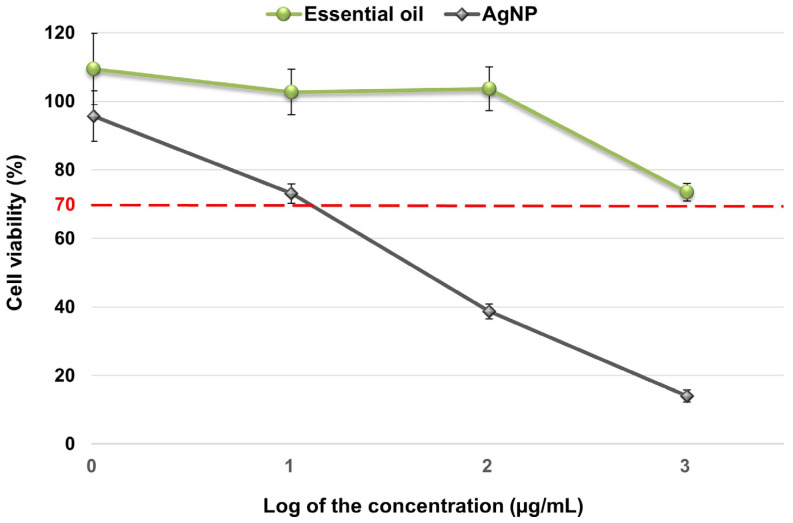
Percentage of cell viability after 24 h of incubation as a function of the logarithm of the concentration (µg/mL) of essential oil and AgNP. Values tested: 0.01 = log 1.024; 1.01 = log 10.24; 2.01 = log 102.4; 3.01 = log 1024. The dashed red line indicates 70% viability. Samples with less than 70% viability were considered cytotoxic.

Results of the cytotoxicity assays of essential oil and AgNP on peripheral blood mononuclear cells after 24 h incubation are shown in Figure 2. The essential oil was found to be non-cytotoxic at the concentrations tested, while AgNP was found to be cytotoxic at a higher concentration of 10.24 µg/mL.

### 2.4. Antimicrobial Activity of Essential Oil and Silver Nanoparticles

**Figure 3:**
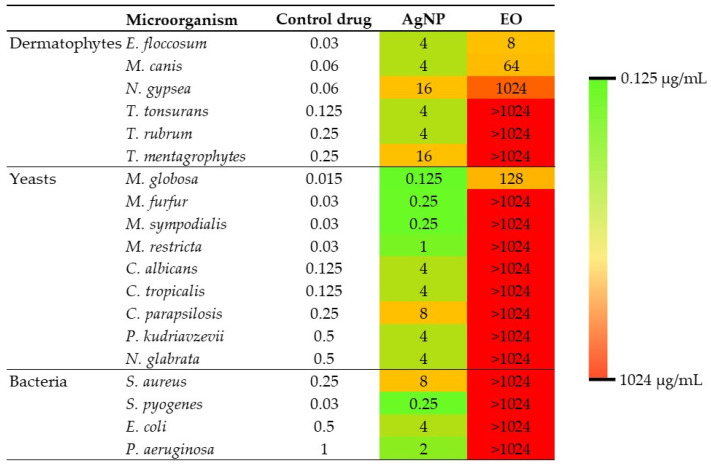
Heat map constructed from the MIC values obtained for the essential oil and AgNP against the tested microorganisms. EO: essential oil. Drug controls: Itraconazole for dermatophytes and yeasts, penicillin for S. pyogenes and gentamicin for other bacteria.

MIC values of essential oil and AgNP against the tested microorganisms are shown in Figure 3.

### 2.5. Synergy Testing

The effect of the interaction between essential oil and AgNP was evaluated against microorganisms that showed a MIC value ≤ 1024 μg/mL for both compounds (Table 2). The test was not performed against bacteria and fungi against which the essential oil was inactive (MIC > 1024 μg/mL).

## 3. Discussion

In recent years, the steady rise of antimicrobial-resistant strains has made combination therapy one of the most widely used tools for the treatment of infections caused by microorganisms. Research is increasingly focused on developing new antimicrobial agents and finding combinations with synergistic effects to improve the spectrum of activity and reduce the dose required, thereby reducing toxic side effects, improving safety and tolerability, and preventing therapeutic failure. In addition, important studies have been published evaluating the effects of the interaction of medicinal plants with clinical drugs for the treatment of various diseases, and of AgNP with antimicrobial agents, several of which have shown synergistic effects [5,14,15,16,17,18]. The present research represents a significant contribution to the field of antimicrobial therapy as, to the best of our knowledge, it is the first report to investigate the synergistic potential of biogenic AgNP and essential oil against causative agents of skin infections.

Essential oils are complex mixtures of volatile plant secondary metabolites, many of which have been shown to have antimicrobial activity [19,20]. In this work, three predominant compounds were identified in the essential oil obtained from *A. australe* leaves collected in northeastern Argentina: germacrene A (24.07%), γ-cadinene (21.47%) and trans-caryophyllene (14.97%). The same compounds were also reported by Morais [21] as major compounds in the essential oil of *A. australe* leaves collected in Rondonia, Brazil, although in different proportions: trans-caryophyllene (16.0%), γ-cadinene (13.0%, 0%) and germacrene A (10.1%). The antimicrobial activity assays showed that *A. australe* essential oil has antifungal activity against *E. floccosum*, *M. canis* and *N. gypsea*, causative agents of dermatophytosis, and against *M. globosa*, the major causative agent of pityriasis versicolor [4]. The observed antifungal activity may be attributed to one or more of the main compounds, which were found to be terpenes. Due to the wide structural variety of terpenes, the antimicrobial mode of action of these compounds has not yet been fully elucidated. However, it is believed that, due to their lipophilic nature, these compounds easily penetrate biological membranes and exert their antimicrobial activity through various mechanisms, including membrane alteration, production of reactive oxygen species and inhibition of essential enzymes, ultimately leading to cell death [22]. Other studies have demonstrated that a metabolic product of caryophyllene, caryophyllene oxide, has been shown to be antifungal against *T. rubrum* and *T. mentagrophytes* [23]. In addition, trans-caryophyllene has been shown to have antibacterial activity against *S. aureus*, *Salmonella typhimurium*, *E. coli* and *Enterococcus faecalis*, as well as a wide range of dental plaque bacteria including *Staphylococcus mutans* and *Streptococcus sobrinus* [24], and significant antiparasitic activity against *Leishmania amazonensis* [25]. Therefore, caryophyllene could be involved in the antifungal activity of the essential oil demonstrated in this work. However, further studies are needed to identify which of the main compounds are responsible for the antifungal activity of *A. australe* essential oil, as no previous reports have been found on the activity of these pure compounds against the microorganisms for which the essential oil has shown antifungal activity.

The green synthesis of AgNP from the aqueous extract of *A. australe* was performed to leverage the reducing and stabilising properties of the plant extract. The choice to use AgNP derived from the aqueous extract instead of *A. australe* essential oil was based on previous findings that the aqueous extract does not exhibit significant antimicrobial activity on its own, but rather acts effectively as a reducing and capping agent for nanoparticle synthesis. This was confirmed in earlier studies where quercetin from the aqueous extract was identified as the main component involved in the nanoparticle coating [26]. By using this method, we ensured the production of stable, biogenic AgNP with desirable antimicrobial properties. Extensive characterisation of the AgNP revealed non-agglomerated spherical nanoparticles with a diameter of 15 ± 3 nm and a strong negative surface charge (zeta potential of −35 ± 6 mV), indicating good colloidal stability. EDX analysis confirmed the purity of the AgNP and the presence of traces of other elements that could be derived from plant compounds present on the surface of the nanoparticles. FTIR analysis showed peaks corresponding to the C=O functional groups of the amide (1640 cm^−1^), the C≡C region (2100 cm^−1^) and the N–H/O–H vibrational region of the amine (3370 cm^−1^), which are similar to the functional groups of AgNP synthesised with quercetin [27]. The results obtained show that the AgNP obtained in this work have similar characteristics to those previously synthesised in terms of size, shape, zeta potential and coating components [26].

The AgNP solution showed strong antimicrobial activity against all bacteria, yeasts and filamentous fungi tested, highlighting its versatility as a broad-spectrum antimicrobial agent. The mechanism of action of biogenic AgNP is not fully understood. However, it is known that they act through multiple pathways that interfere with the cellular functions of microorganisms. Furthermore, their antimicrobial efficacy is highly dependent on the size, shape and coating of the nanoparticles [8].

The in vitro toxicity of the essential oil and AgNP solution was evaluated on peripheral blood mononuclear cells to anticipate possible adverse effects in humans. Our results showed that the essential oil had no toxic effects at the concentrations evaluated and the AgNP solution was found to be cytotoxic at concentrations higher than 10.24 μg/mL. These results suggest that non-cytotoxic concentrations of these nanoparticles can be used to inhibit microbial growth, except in the case of *N. gypsea* and *T. mentagrophytes*. By working with non-toxic concentrations of at least one of the components, either AgNP or essential oil, we ensure that, upon finding a synergistic antimicrobial effect, the concentration of the component that exhibits toxicity could be reduced to a non-toxic level. This allows us to identify the most effective and safe combinations against susceptible microorganisms. However, it is important to highlight that further studies are needed to confirm the safety of the combined use of both compounds.

It was possible to evaluate the effect of the interaction between the *A. australe* essential oil and biogenic AgNP against the microorganism species that both compounds demonstrated to be active (MIC ≤ 1024 μg/mL). Our decision not to study compounds with MIC > 1024 μg/L is based on several aspects: avoiding false positives due to inhibition of microbial growth by using very high and potentially toxic concentrations instead of true effective antimicrobial activity; overcoming the technical difficulties of evaluation caused by the turbidity and low solubility of the essential oil at high concentrations, which makes it difficult to read the MIC accurately; and working with compounds that have proven and safe activity [11]. This methodological approach allows us to obtain reliable and therapeutically relevant results, significantly contributing to the field of antimicrobial research. In this work, a synergistic effect was observed against *M. canis*, *N. gypsea* and *M. globosa*, and no antagonistic effect was observed in any of the trials. Furthermore, the combination allowed the use of a non-cytotoxic concentration of AgNP capable of inhibiting *N. gypsea* growth. Therefore, the combined use of essential oil and AgNP represents a potential alternative for the treatment of pityriasis versicolor and dermatophytosis. Although both compounds were active independently, no interaction was observed against *E. floccosum*. The fold decrease (fd) values shown in Table 2 indicate how many times the MIC of a compound is reduced when used in combination with another compound compared to when used alone. This value is an important measure that helps to understand the enhanced efficacy of compounds when used together [28]. The results show that in microorganisms where a synergistic effect was observed from the combined use of AgNP and essential oil, the concentration of both compounds was reduced by 4 to 8 times, thereby improving their antimicrobial efficacy. These findings highlight the potential of combining AgNP and essential oils as a strategy to enhance antimicrobial activity against certain pathogens that cause skin infections. However, it is important to note that the interaction between two compounds can alter their toxicity profiles, making it necessary to conduct further studies to understand the underlying mechanisms of this synergy and to explore its application in in vivo models.

In conclusion, the results obtained in this work suggest a high antimicrobial activity of the biogenic AgNP, but the combination with *A. australe* essential oil could overcome the individual limitations, mainly the AgNP toxicity, and enhance their antimicrobial potential, representing an innovative strategy for the treatment of skin infections. Furthermore, this work highlights the importance of promoting the study of interactions between metallic nanoparticles and products of natural origin in order to obtain plant-based formulations with alternative therapeutic potential for the treatment of skin infections. This would prevent the emergence of resistant strains by reserving drugs for clinical use in complicated infections.

Future studies should focus on optimising these combinations and evaluating their efficacy and safety using in vivo models, which could open up new avenues for the treatment of skin infections and other infectious diseases.

## 4. Materials and Methods

### 4.1. Plant Material

The leaves of *A. australe* were harvested by hand and dried at room temperature, protected from sunlight. The plant material was collected in Corrientes, Argentina (27°33′42.0″ S 58°43′35.9″ W) and taxonomically identified by the Instituto de Botánica del Nordeste, Universidad Nacional del Nordeste (UNNE), Argentina. A reference specimen (IMR-H-2017-18) is deposited in the herbarium of the Instituto de Medicina Regional (IMR), UNNE, Argentina. The plant material was collected in accordance with previously established recommendations [11] and in compliance with relevant institutional, national and international guidelines and legislation.

### 4.2. Acanthospermum Australe Essential Oil

The essential oil was obtained by hydrodistillation of 200 g of the plant material for 4 h using a Clevenger apparatus. The oil was dried with anhydrous sodium sulphate (Cicarelli, San Lorenzo, Santa Fe, Argentina) and stored in a caramel-coloured glass bottle in a nitrogen atmosphere at 4 °C until further use.

### 4.3. GC–MS Analysis

For essential oil GC–MS analyses, a non-polar HP-5MS (95%-dimethyl-5%-diphenylpolysiloxane; Agilent Technologies, Walt and Jennings Scientific, Wilmington, DE, USA) cross-linked capillary column (30 m × 0.25 mm i.d. × 0.25 μm film thickness) was attached to a Shimadzu GC–MS QP2020 Ultra (Shimadzu Corporation, Kyoto, Japan). The experimental conditions for pure essential oil analysis were as follows: injector, interface and ion source, 280 °C; temperature program: 40 °C (4 min), 40–180 °C at 4 °C min^−1^, 180 °C (2 min), 180–280 °C at 10 °C min^−1^, 280 °C (10 min). The ionisation energy was 70 eV; injection size, 1 μL (split ratio 25:1) and helium (99.9999%; Linde, Munich, Germany) was used as carrier gas at a flow rate of 1.0 mL min^−1^. Mass range: 35–400 *m*/*z*.

The constituents of the essential oil were identified by comparison of their mass spectra with those stored in GC–MS databases [29,30]. The linear retention index (LRI) was calculated for each identified compound in relation to a series of C8–C20 n-alkanes (Sigma-Aldrich, St. Louis, MO, USA) and then compared with the databases [30,31,32]. The content of the eluted components was expressed as a percentage of the relative area of the peaks.

### 4.4. Green Synthesis of Silver Nanoparticles

Initially, an aqueous extract of *A. australe* was obtained by maceration of 25 g of crushed plant material with 1000 mL of sterile, double-distilled water in a hermetically sealed container under constant stirring. The maceration process was continued for 12 h at a controlled temperature of 40 °C using a thermostatically controlled orbital shaking incubator at 100 rpm. The extract was then filtered first through Whatman No. 1 filter paper and then through a Millipore filter with a porosity of 0.22 μm, followed by lyophilisation. The resulting dry extract was stored at 4 °C in a sterile amber glass bottle, hermetically sealed and protected from light until use.

Secondly, the lyophilised aqueous extract was resuspended in sterile deionised water at a concentration of 50 mg lyophilised extract per millilitre and adjusted to pH 9.5 with 0.1 N NaOH. Then 10 mL of this suspension was added to 190 mL of 1 mM AgNO_3_ solution. The reaction was carried out at 95 °C for 15 min. The AgNP obtained were purified by centrifugation and resuspension (3 times) of the sediment in sterile deionised water at 15,000 rpm for 20 min.

### 4.5. Characterisation of Silver Nanoparticles

The synthesised AgNP solution was characterised by UV–visible spectrophotometry (Multiskan Go, Thermo Fischer Scientific, Vantaa, Finland), transmission electron microscopy (TEM; JEOL, Tokyo, Japan; JEM-2100), energy-dispersive X-ray spectroscopy (EDX) and Fourier transform infrared spectroscopy (FTIR; Madison, WI, USA; Nicolet 670). In addition, the hydrodynamic size, polydispersity index and zeta potential of the AgNP solution were determined by dynamic light scattering (DLS) using a Malvern Zetasizer Nanoseries compact dispersion spectrometer (Malvern, UK).

### 4.6. Cytotoxicity Assay

Cytotoxicity of the AgNP solution and essential oil was tested on peripheral blood mononuclear cells using the method described previously [26]. Following the guidelines of ISO 10.993-5 [33], four concentrations covering a wide range were tested. The maximum concentration tested was 1024 μg/mL, which corresponded to the level above which the essential oil was considered inactive against a specific microorganism. The percentage of cell viability was determined by comparison with the negative control (untreated cells). Samples with less than 70% viability were considered cytotoxic [33]. All cytotoxicity assays were repeated 6 times for each concentration.

### 4.7. Microorganisms

A total of 19 microorganisms were tested: *Staphylococcus aureus* ATCC 29213, *Pseudomonas aeruginosa* ATCC 27853, *Escherichia coli* ATCC 25922, *Streptococcus pyogenes* IMR-B 713, *Malassezia furfur* CBS 7019, *Malassezia sympodialis* CBS 7222, *Malassezia globosa* CBS 7986, *Malassezia restricta* IMR-ML 454, *Candida albicans* ATCC 90028, *Candida tropicalis* ATCC 750, *Pichia kudriavzevii* (formerly *Candida krusei*) ATCC 6258, *Candida parapsilosis* ATCC 22019, *Nakaseomyces glabrata* (formerly *Candida glabrata*) ATCC 2001, *Microsporum canis* IMR-MD 5, *Nannizzia gypsea* IMR-MD 16, *Epidermophyton floccosum* IMR-MD 26, *Trichophyton tonsurans* IMR-MD 8, *Trichophyton rubrum* IMR-MD 3 and *Trichophyton mentagrophytes* IMR-MD 7. All microorganisms were obtained from the culture collection of the IMR, UNNE, Argentina.

### 4.8. Antimicrobial Activity

To evaluate the antimicrobial activity of the essential oil and AgNP solution, the minimum inhibitory concentration (MIC) was determined using the broth microdilution method according to CLSI M27 protocols for yeasts [34], CLSI M38 for filamentous fungi [35] and CLSI M07 for bacteria [36]. The following drugs were used as controls: itraconazole (Sigma-Aldrich) for fungi, penicillin (Sigma-Aldrich) for *S. pyogenes* and gentamicin (Sigma-Aldrich) for *E. coli*, *S. aureus* and *P. aeruginosa*.

For the essential oil and AgNP solution, the MIC was defined as the lowest concentration that inhibited 100% of microbial growth compared to the growth control. The antimicrobial activity criterion established in a previous work was used [11]: essential oils, being a complex mixture of compounds, are considered active if the MIC ≤ 1024 μg/mL and inactive at higher values, whereas pure compounds such as metallic nanoparticles are considered active if the MIC ≤ 256 μg/mL and inactive at higher values.

All antimicrobial activity assays were performed in duplicate. MIC values are expressed in μg/mL. The concentration of the AgNP solution was determined by measuring its dry weight.

### 4.9. Synergy Testing

The checkerboard titration method was used to evaluate the synergistic antimicrobial effect as a result of the interaction between *A. australe* essential oil and AgNP synthesised from the aqueous extract of *A. australe* [5,37]. Fractional inhibitory concentration index (FICi) values were interpreted according to Odds [38] as synergism (FICi ≤ 0.5), no interaction (FICi > 0.5–4.0) and antagonism (FICi > 4.0).

## Figures and Tables

**Table 1:** Chemical composition of essential oil from *A. australe* leaves. Compounds are listed in order of elution. Retention time (RT). Linear retention index (LRI).

RT	LRI	Identified Compound Name	Area (%)
6.811	933	α-Thujene	0.01
7.204	936	α-Pinene	5.53
7.724	950	Camphene	0.1
8.935	973	Sabinene	0.15
9.088	978	β-Pinene	1.26
9.682	986	6-Methyl-5-heptene-2-one	0.03
9.908	989	Myrcene	0.61
11.23	1017	α-Terpinene	0.02
11.775	1024	para-Cymene	1.99
12.092	1030	Limonene	4.28
12.61	1038	(Z)-β-Ocimene	0.03
13.22	1048	(E)-β-Ocimene	0.07
13.54	1038	2,6-Dimethyl-5-heptenal	0.03
13.778	1060	γ-Terpinene	0.03
15.119	1192	Myrtanal	0.01
15.637	1087	Terpinolene	0.04
16.52	1099	Linalool	0.04
16.814	1103	Nonanal	0.02
17.493	1119	Octen-1-ol, acetate	0.26
18.288	1110	3-Octyl acetate	0.02
18.578	1134	Limonene oxide <cis->	0.01
18.712	1134	Mentha-2,8-dien-1-ol <cis-, para->	0.01
18.91	1138	Limonene oxide <trans->	0.02
19.354	1149	Isopulegol	0.04
20.008	1151	Menthone	0.03
20.314	1154	Citronellal	3.19
23.736	1205	Decanal	0.02
24.478	1217	trans-Carveol	0.02
25.35	1228	Citronellol	0.06
25.713	1234	Thymol methyl ether	0.17
26.13	1242	Carvone	0.04
27.139	1255	Geraniol	0.09
28.176	1242	Neral	0.02
29.421	1290	Thymol	0.04
29.815	1300	Carvacrol	0.14
31.567	1329	Silphiperfol-5-ene	0.07
32.617	1337	δ-Elemene	0.56
33.842	1378	Silphiperfol-6-ene	0.34
35.238	1380	β-Patchoulene	0.44
35.606	1384	β-Bourbonene	0.04
36.08	1433	β-Copaene	0.1
36.242	1390	β-Elemene	0.59
38.324	1420	trans-Caryophyllene	14.97
38.701	1387	β-Cubebene	1.81
39.135	1494	Bicyclogermacrene	0.15
39.598	1481	Germacrene D	0.57
40.248	1453	α-Humulene	4.34
42.502	1515	γ-Cadinene	21.47
43.217	1501	Germacrene A	24.07
44.921	1523	δ-Cadinene	1.15
45.431	1550	(E)-γ-Bisabolene	0.83
51.784	1651	Intermedeol<neo->	0.54
54.382	1681	Germacra-4(15),5,10(14)-trien-1-α-ol	0.97
63.13	1849	Phytone	0.97
69.397	1970	Palmitic acid	2.16
71.684	2116	Phytol	1.96
71.98	2134	Linoleic acid	0.73
72.248	2200	Stearic acid	0.16

**Table 2:** Fractional inhibitory concentration index (FICi) values obtained and interpretation of AgNP–essential-oil interaction.

Microorganism	AgNP			EO			FICi	Interpretation
	MIC_c_	FIC_AgNP_	fd	MIC_c_	FIC_EO_	fd		
*E. floccosum*	2	0.5	2×	4	0.5	2×	1	No interaction
*M. canis*	1	0.25	4×	8	0.125	8×	0.375	Synergism
*N. gypsea*	2	0.125	8×	256	0.25	4×	0.375	Synergism
*M. globosa*	0.03	0.25	4×	32	0.25	4×	0.5	Synergism

FICi value was determined using the following formula: FICi = FIC_AgNP_ + FIC_EO_. FIC_AgNP_ and FIC_EO_ values were determined using the following formula: FIC = MIC_c_/MIC_alone_. MIC_c_ is the minimum inhibitory concentration of the compound in combination with the other compound. MIC_alone_ is the minimum inhibitory concentration of the compound alone. The fold decrease (fd) describes how much the MIC of the compound in combination was reduced compared to the MIC of the same compound alone (MIC values of the compounds individually are shown in Figure 3).

## Data Availability

The data that support the findings of this study are available from the corresponding author upon reasonable request.

## Supplementary Materials

The following supporting information can be downloaded at: https://www.mdpi.com/article/10.3390/antibiotics13070674/s1, Figure S1: GC-MS chromatogram of the essential oil obtained from the leaves of *A. australe*.
