# Supplementary material for: Synergistic Antimicrobial Activity of Biogenic Silver Nanoparticles and Acanthospermum australe Essential Oil against Skin Infection Pathogens
Source: Antibiotics (Basel). 2024 Jul 20;13(7):674. doi: 10.3390/antibiotics13070674 (PMC11274195; doi:10.3390/antibiotics13070674)
Supplement: Supplementary file 1 [file antibiotics-13-00674-s001.zip › antibiotics-3054229-supplementary.pdf]

Supplementary Materials

Synergistic Antimicrobial Activity of Biogenic Silver Nanoparticles and *Acanthospermum australe* Essential Oil against Skin Infection Pathogens

Javier Mussin * and Gustavo Giusiano

Instituto de Medicina Regional, Universidad Nacional del Nordeste, Consejo Nacional de Investigaciones Científicas y Tecnológicas (CONICET), 3500 Resistencia, Argentina;
gustavogiusiano@yahoo.com.ar
* Correspondence: javiermussin@conicet.gov.ar

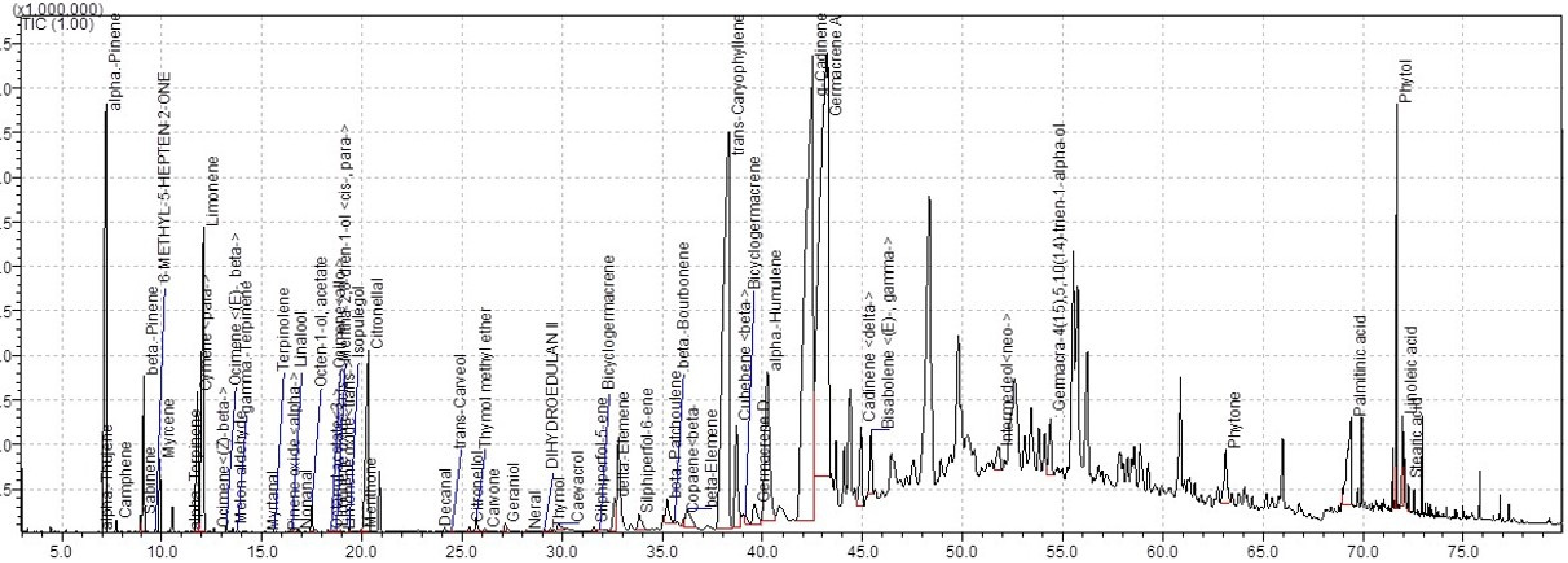

Figure S1. GC-MS chromatogram of the essential oil obtained from the leaves of *A. australe*
